# Multimodel magnetic resonance imaging of mass-forming autoimmune pancreatitis: differential diagnosis with pancreatic ductal adenocarcinoma

**DOI:** 10.1186/s12880-021-00679-0

**Published:** 2021-10-15

**Authors:** Huihui Jia, Jialin Li, Wenjun Huang, Guangwu Lin

**Affiliations:** 1 Department of Radiology, Huadong Hospital Affiliated to Fudan University, 221 West Yanan Road, Shanghai, 200040 China; 2 Department of General Surgery, Center of Pancreaticobiliary Disease, Huadong Hospital Affiliated to Fudan University, Shanghai, China

**Keywords:** Magnetic resonance imaging, Autoimmune pancreatitis, Pancreatic ductal adenocarcinoma, Diffusion-weighted imaging

## Abstract

**Purpose:**

To assess the value of the multimodel magnetic resonance imaging (MRI), including unenhanced images, dynamic contrast-enhanced MRI (DCE-MRI), MR-cholangiopancreatography (MRCP), and diffusion-weighted imaging (DWI), in differentiation of mass-forming autoimmune pancreatitis (AIP) from pancreatic ductal adenocarcinoma (PDAC).

**Methods:**

Twelve patients with mass-forming AIP and 30 with PDAC were included. All patients underwent unenhanced MRI, DCE-MRI, DWI, and MRCP. Relevant values including sensitivity and specificity of the imaging features and their diagnostic performance for predicting mass-forming AIP were analyzed.

**Results:**

Several statistically significant MR findings and quantitative indexes differentiating mass-forming AIP from PDAC, including multiplicity, irregularity or conformation, capsule-like rim enhancement, absence of internal cystic or necrotic portion, homogeneous enhancement during pancreatic, venous, and delayed phases, skipped stricture or stricture of MPD, absence of side branch dilation, maximum upstream MPD diameter < 2.4 mm, Contrast_UP_ > 0.739, Contrast_AP_ > 0.710, Contrast_PP_ > 0.879, and Contrast_VP_ or Contrast_DP_ > 0.949, indicated mass-forming AIP (*P* < 0.05). The apparent diffusion coefficient (ADC) value was also significantly lower in mass-forming AIP compared to that in PDAC (*P* = 0.006). The cutoff value of ADC for distinguishing mass-forming AIP from PDAC was 1.099 × 10^−3^ mm^2^/s.

**Conclusion:**

Multimodel MRI, including unenhanced MRI, DCE-MRI with DWI and MRCP can provide qualitative and quantitative information about mass-forming AIP characterization. Multimodel MRI are valuable for differentiating mass-forming AIP from PDAC.

## Background

Autoimmune pancreatitis (AIP) is a distinct type of chronic fibroinflammatory pancreatitis characterized by abundant lymphoplasmacytic infiltration, interstitial fibrosis, and elevated serum levels of IgG4 [1, 2]. The etiology of AIP is yet unclear [2–4]. According to the different clinicopathological characteristics, AIP is now divided into type 1 and type 2 subtypes [2, 5]. Type 1 is an IgG4-related systemic disease that can have extra-pancreatic involvement [5, 6]. Type 2 is characterized histologically by idiopathic duct-centric pancreatitis [6]. Type 2 is not associated with either serum IgG4 elevation or extrapancreatic involvement [5].

In the event of diffuse involvement, the enlarged pancreas manifests a “sausage-like” appearance, which is different from that of pancreatic ductal adenocarcinoma (PDAC) [7, 8]. Mass-forming AIP accounts for approximately 28–41% of all cases of AIP [9, 10]. Mass-forming AIP sometimes is difficult to differentiate from PDAC as the two diseases have the overlapping clinical and radiological features. AIP responds dramatically to steroid therapy, while PDAC is curable only by surgical resection [1, 11]. Furthermore, the differentia between the two types of diseases is crucial as treatment and prognosis are different.

In this study, the diagnostic dilemma of differentiating between these two entities is discussed. In addition, we review the characteristics of each condition by multimodel magnetic resonance imaging (MRI), including unenhanced MRI, dynamic contrast-enhanced MRI (DCE-MRI), MR-cholangiopancreatography (MRCP), and diffusion-weighted imaging (DWI) findings, for a better understanding of the two conditions.

## Methods

### Patients

**Fig. 1:**
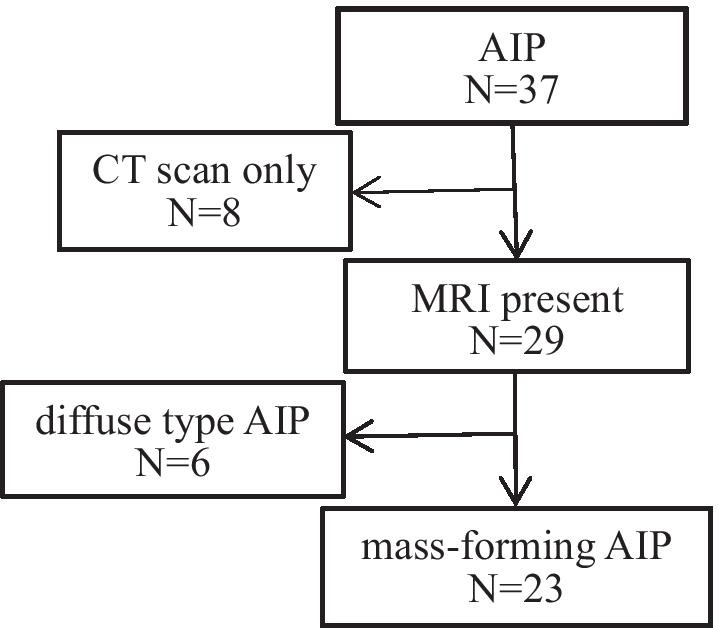
Flow diagram of study enrollment population

This retrospective study was approved by the Ethics Committee of Fudan University and all the procedures were conducted in accordance with the Declaration of Helsinki of 1996. From our institution’s medical database between January 2015 and February 2020, 37 patients diagnosed with AIP were found in the study based on the diagnostic criteria [12–14]. Twenty-five patients with AIP were excluded: 8 had only undergone computed tomography (CT) scan, 10 with only unenhanced MRI images without dynamic enhancement, and 1 with only dynamic enhancement without unenhanced MRI images, while the remaining 6 patients were diffuse type AIP. Finally, 12 patients with mass-forming AIP, according to the histopathological confirmation at surgery or biopsy (n = 7) or by the combination of elevated serum IgG4 level, typical imaging features, and response dramatically well to steroid therapy (n = 5), were included in this study. Additionally, data from 30 PDAC patients with pathologically confirmed between January 2019 and February 2020 were also analysed (Fig. 1).

### MRI techniques

MRI was performed using a 3.0 T scanner (Magnetom Skyra, Siemens, Erlangen, Germany) with an 8-channel body-phased array coil. T2-weighted half-Fourier acquisition single-shot turbo spin-echo (HASTE) sequence, navigator-triggered fat-suppressed T2-weighted turbo spin-echo sequence, MRCP images, breath-hold T1-weighted Dixon vibe sequence, DWI and dynamic imaging with a three-dimensional volumetric interpolated breath-hold examination (3D-VIBE) sequence were included (Table 1). 3D-VIBE sequence was obtained before (unenhanced phase) and 20 s (arterial phase, AP), 35 s (pancreatic phase, PP), 50 s (portal venous phase, VP), and 3–6 min (delayed phase, DP) after intravenous administration of gadopentetate dimeglumine (Magnevist; Bayer Schering Pharma). The contrast agent was injected intravenously as a bolus (3.0 mL/s) at a dose of 0.1 mmol/kg followed by flushing with 20 mL saline using a power injector. The apparent diffusion coefficient (ADC) value was calculated with b values 50 and 800 s/mm^2^, respectively. The average interval between MRI and surgery was 10 days (range, 1–30 days).

**Table 1:** MRI sequences and parameters

	T2WI		T1WI	Thick slab single slice MRCP	Thin slab multislice MRCP	3D VIBE	DWI
	HASTE	RT-TSE					
TR/TE (ms)	1600/95	3000/79	4/1.29	4500/979	1150/252	3.55/1.27	4700/66
FA (°)	150°	120°	9°	180°	121°	13°	–
Section thickness (mm)	5	5	3	45	4	2.5	6
Matrix	320 × 256	320 × 256	320 × 256	512 × 320	512 × 409	320 × 256	192 × 153
FOV (cm)	32–38	32–38	32–38	30	32	32–38	32–38
Acquisition time (s)	60	150	16	5	20	18	198

*T2WI* T2-weighted images, *T1WI* T1-weighted images, *MRCP* magnetic resonance cholangiopancreatography, *3D VIBE* three-dimensional volumetric interpolated breath-hold examination, *DWI* diffusion-weighted imaging, *HASTE* half-Fourier acquisition single-shot turbo spin-echo, *RT-TSE* respiratory-triggered turbo spin-echo, *TR* repetition time, *TE* echo time, *FA* flip angle, *FOV* field of view

### Image analysis

The MR images were reviewed independently by two radiologists (GWL and HHJ with 15 and 7 years of experience in abdominal imaging, respectively) who were blinded to the results aside from the diagnosis including AIP and PDAC. In case of disagreements, the discussion was continued until a final consensus was reached.

Items analyzed for each mass included the number of masses, location (uncinate, head or neck, body or tail), margin (indistinct, distinct), contour (focal and diffuse enlargement, focal protrusion, or no bulging), shape (oval or round, irregular, or geographic), capsule-like rim, internal cystic or necrotic component, upstream pancreatic atrophy, peripancreatic infiltration, vascular invasion, lymphadenopathy, T2WI signal intensity (SI) (hypointense, isointense, and hyperintense), T1WI SI, DWI SI, and homogeneity of enhancement or SI in AP, PP, VP, and DP (homogeneous, heterogeneous).

In addition, the MRCP findings were analysed to assess the potential existence of abnormalities of the common bile duct (CBD) and pancreatic ducts. The following characteristics were analyzed: complete obstruction of CBD/main pancreatic duct (MPD), the stricture or skipped stricture of CBD/MPD, the side-branch dilation, and the presence of penetrating duct sign. The penetrating duct sign indicates the MPD penetrating the mass. In the case of multiple lesions in one patient, an image analysis of each lesion characteristic was performed for that patient.

Quantitative analysis of the mass enhancement contrast in AIP and PDAC was performed by another reviewer (WJH, 6 years of experience) who did not participate in the qualitative image analysis. A region-of-interest (ROI) was drawn to assess SI of the pancreatic mass and non-mass adjacent pancreatic parenchyma (NAP) on DCE-MRI. The ROI was lesion-size-dependent for the pancreatic mass. In NAP, the ROI was placed downstream to the mass to exclude any potential obstructive pancreatitis-related SI change. Three measurements were performed for the same image in each phase. The average SI values were calculated to obtain the mass enhancement contrast at each phase: Contrast = SI_mass_/SI_NAP_.

Furthermore, lesion diameter and ADC were measured for quantitative analysis. An ROI was drawn in areas of the pancreatic mass and the NAP for the measurement of ADC, devoid of cystic or necrotic portions. Subsequently, the normalized ADC was calculated: ADC_mass_/ADC_NAP_, where ADC_mass_ is the ADC of the pancreatic mass and ADC_NAP_ is the ADC of the NAP. The ADC maps were available for 16 masses of mass-forming AIP and 30 masses of PDAC. Also, the maximum upstream MPD diameter was also measured in all patients.

### Statistical analysis

Pearson’s chi-square test and Fisher’s exact test were performed to compare categorical variables between AIP and PDAC groups. Student’s t-test was performed for continuous variables. Interobserver variability regarding the qualitative parameters was assessed by using Kappa test. The strength of agreement was assessed as follows: k values < 0.20 indicated poor agreement; k values of 0.21–0.40 fair agreement; k values of 0.41–0.60 moderate agreement; k values of 0.61–0.80 good agreement; k values of 0.81–1.00 excellent agreement. Student’s t-test was used to compare the mass enhancement contrast at each phase of DCE-MRI between the two groups. Receiver operating characteristic (ROC) was employed to analyze the diagnostic performance of the lesion enhancement contrast at each phase in distinguishing AIP from PDAC. The optimal cutoff point of the mass enhancement contrast was determined to maximize both the sensitivity and specificity (i.e., Youden index). The sensitivity and specificity of each imaging finding was calculated. MedCalc version 19.1 software (MedCalc Software, Mariakerke, Belgium) and SPSS version 22.0 (IBM SPSS Inc., Chicago, IL, USA) were used for statistical analysis. *P* < 0.05 indicated statistical significance.

## Results

### Qualitative analysis of MRI features

**Fig. 2:**
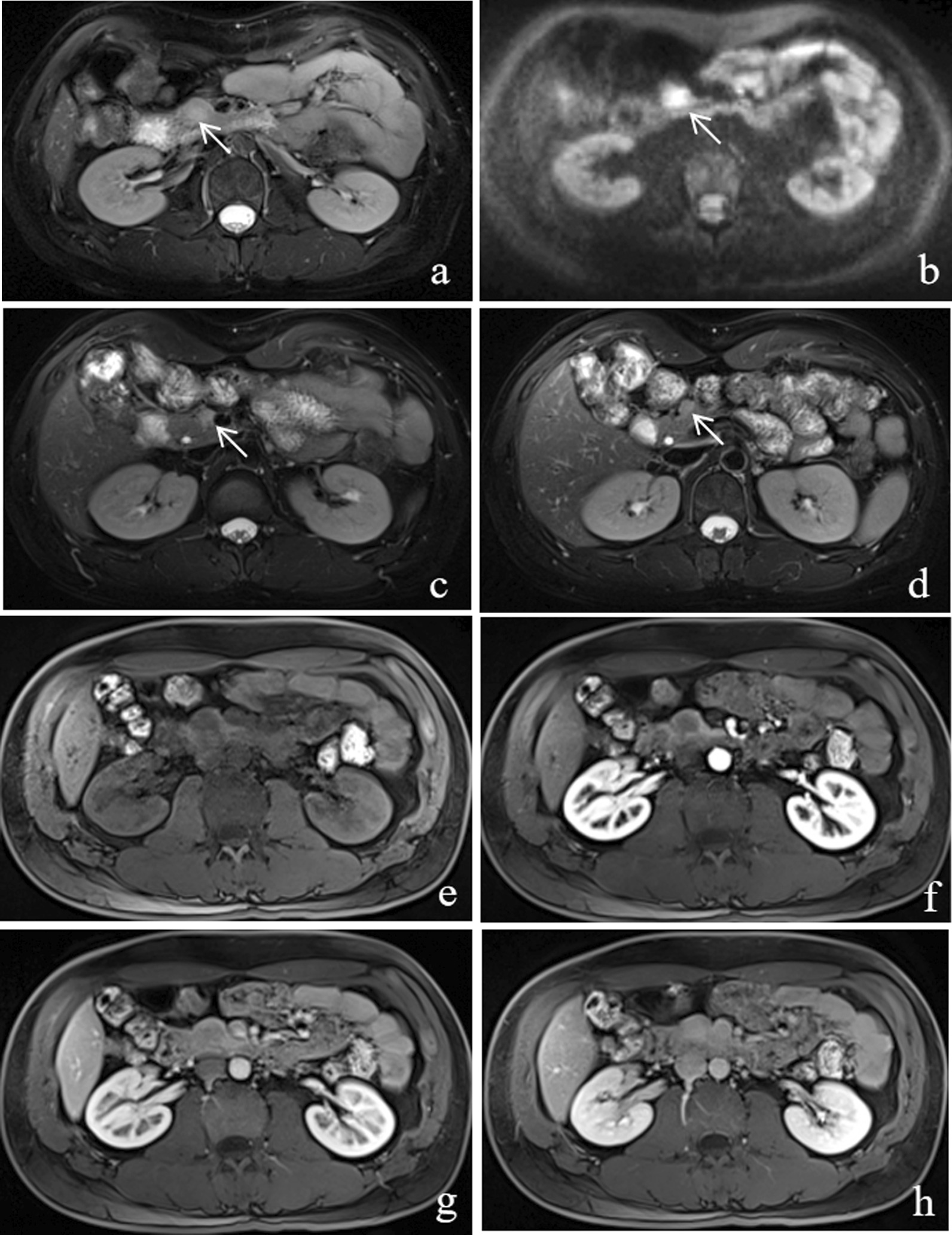
A 30-year-old woman had mass-forming AIP with multiple masses in the pancreas. **a** T2WI, a round-shaped mass located in the pancreatic head (arrow) is observed as hyperintense. **b** On DWI (b = 800 s/mm^2^), the mass appears hyperintense compared to the pancreatic parenchyma (arrow). Another two small, round-shaped, hyperintensity masses are located in the pancreatic head (**c**) and neck (**d**) (arrows). **e** Unenhanced T1-weighted MR image exhibits a round hypointense nodule in the head of the pancreas. **f** On the AP image, the mass demonstrates a well-defined, obvious hypointensity. **g** On the PP image, the mass shows subtle hypo- or isointensity. **h** The DP image demonstrates the homogeneous hyperintensity of the mass

**Fig. 3:**
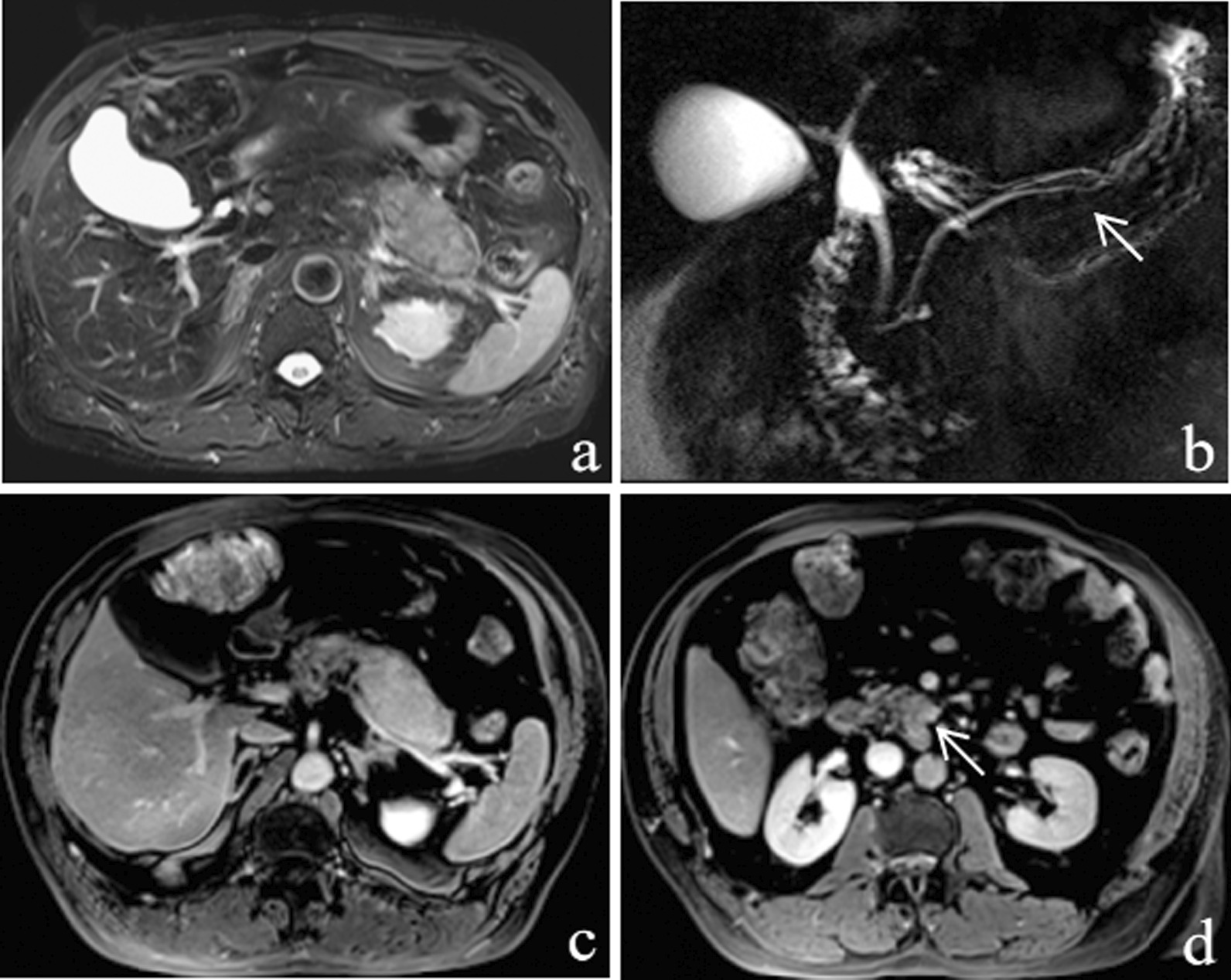
A 63-year-old man had mass-forming AIP with two masses in the pancreas. **a** T2WI, an oval-shaped mass located in the pancreatic tail, is clearly observed as hyperintense with an indistinct margin. In addition, a hypointense rim is seen surrounding the pancreas. **b** MRCP image shows stenosis of the MPD of the pancreatic tail (penetrating duct sign) (arrow). **c** On the DP image, the mass shows hyperintensity with a peripancreatic enhancing rim (capsule-like rim enhancement). **d** A second area of lesion is observed in the pancreatic uncinate process (arrow). The area appears round and heterogeneously hyperintense

For qualitative analysis, MRI characteristics are summarized in Table 2. Multiple pancreatic masses were significantly more prevalent in AIP (3 patients with two lesions and 1 with three lesions) than in PDAC without multiple lesions (*P* = 0.004) (Figs. 2, 3). Mass-forming AIP (9/17, 52.9%) constantly exhibited an irregular or geographical morphology, while PDAC manifested either an oval or round shape (27/30, 90.0%) (*P* = 0.004). The sign of capsule-like rim was presented exclusively in AIP patients (6/17, 35.3%), while internal cyst or necrotic portion (*P* = 0.008) was commonly observed in the PDAC patients.

**Table 2:** MRI findings

	AIP (*N* = 17 masses in 12 patients)	*k*	PDAC (*N* = 30)	*k*	*P* value
Number of masses		0.824		1	0.004
1	8/12		30/30		
2	3/12		0/30		
3	1/12		0/30		
Location		0.904		0.820	0.343
Uncinate	3/17		2/30		
Head or neck	6/17		16/30		
Body or tail	8/17		12/30		
Margin		0.876		1	0.214
Indistinct	7/17		18/30		
Distinct	10/17		12/30		
Contour		1		0.776	0.173
Focal and diffuse enlargement	3/17		4/30		
Focal protrusion	9/17		23/30		
No bulging	5/17		3/30		
Shape		0.881		0.839	0.004
Oval or round	8/17		27/30		
Irregular or geographical	9/17		3/30		
Capsule-like rim	6/17	0.866	0/30	1	0.001
Internal cystic or necrotic portion	0/17	1	10/30	0.923	0.008
Upstream pancreatic atrophy	2/12	1	10/30	0.700	0.453
Peripancreatic infiltration	1/12	1	12/30	0.789	0.067
Vascular invasion	2/12	0.625	10/30	0.923	0.453
Lymphadenopathy	2/12	1	3/30	1	0.613
T2WI signal intensity		0.767		1	0.128
Isointense	3/17		1/30		
Hyperintense	14/17		29/30		
T1WI signal intensity		0.767		1	1.000
Hypointense	16/17		29/30		
Isointense	1/17		1/30		
DWI signal intensity		0.821		0.902	1.000
Hyperintense	14/17		24/30		
Isointense	3/17		6/30		
Signal intensity and enhancement of mass					
The arterial phase					
Hypointense	15/17	1	29/30	1	0.544
Isointense	2/17		1/30		
Hyperintense	0/17		0/30		
Homogeneous	4/17	0.821	2/30	1	0.170
Heterogeneous	13/17		28/30		
The pancreatic phase					
Hypointense	9/17	0.901	25/30	0.621	0.017
Isointense	3/17		4/30		
Hyperintense	5/17		1/30		
Homogeneous	8/17	0.764	2/30	1	0.002
Heterogeneous	9/17		28/30		
The venous phase					
Hypointense	2/17	1	23/30	0.918	< 0.001
Isointense	2/17		4/30		
Hyperintense	13/17		3/30		
Homogeneous	12/17	0.866	2/30	1	< 0.001
Heterogeneous	5/17		28/30		
The delayed phase					
Hypointense	1/17	1	15/30	0.725	0.001
Isointense	1/17		4/30		
Hyperintense	15/17		11/30		
Homogeneous	13/17	0.850	2/30	1	< 0.001
Heterogeneous	4/17		28/30		

Numbers represent the number of patients

**Fig. 4:**
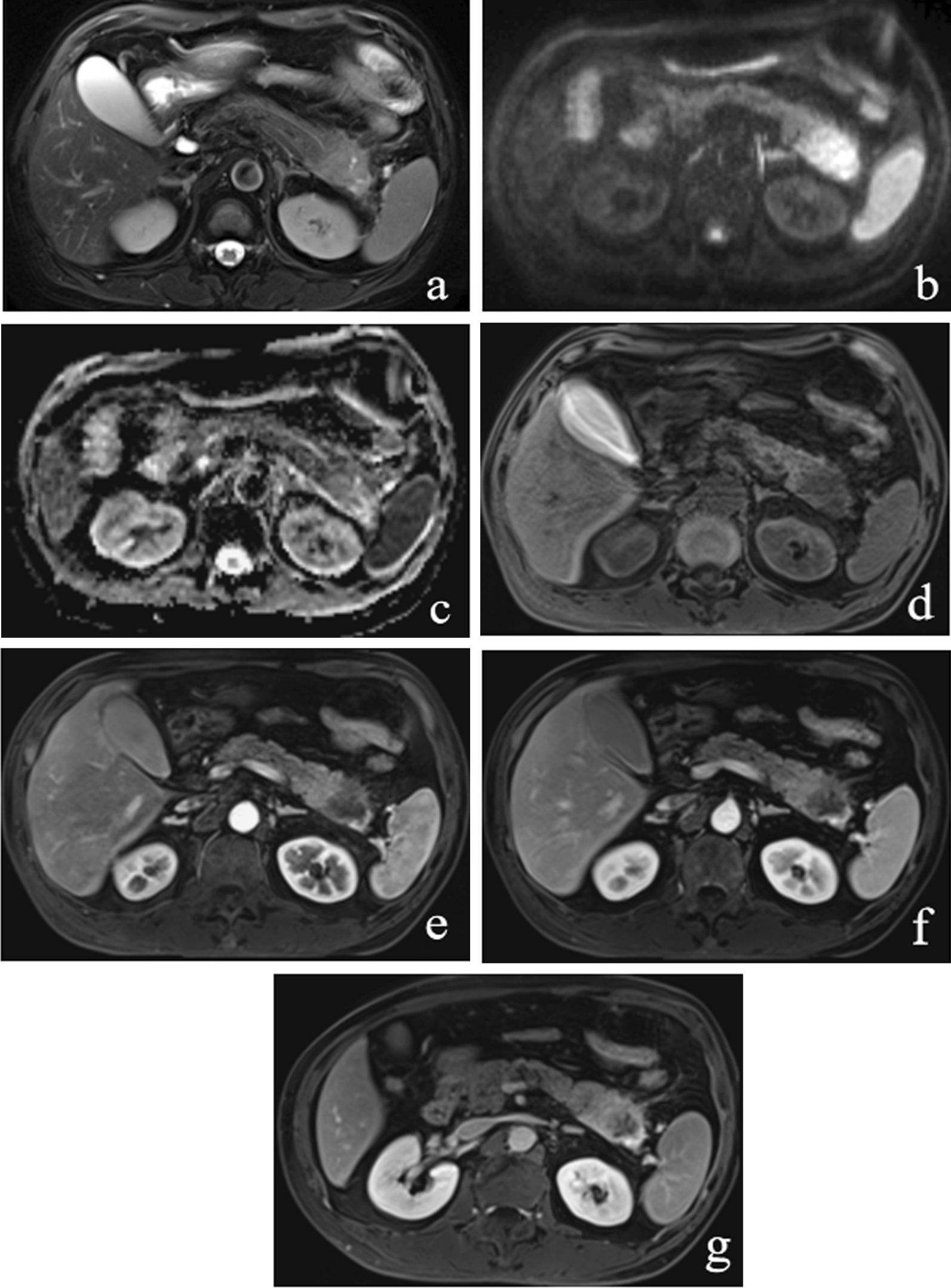
A 62-year-old man had PDAC. **a** T2WI shows an oval-shaped mass located in the pancreatic tail that is visible as hyperintense with heterogeneity. **b** On DWI (b = 800 s/mm^2^) and ADC map (**c**), the pancreatic mass is clearly defined as hyperintense and hypointense, respectively. The pancreatic mass in the tail appears hypointense on both unenhanced (**d**) and (**e**) PP images. During the VP (**f**) and DP (**g**) imaging, the mass appears mild or obviously hyperintense, but the central area maintains hypointensity all the time

**Fig. 5:**
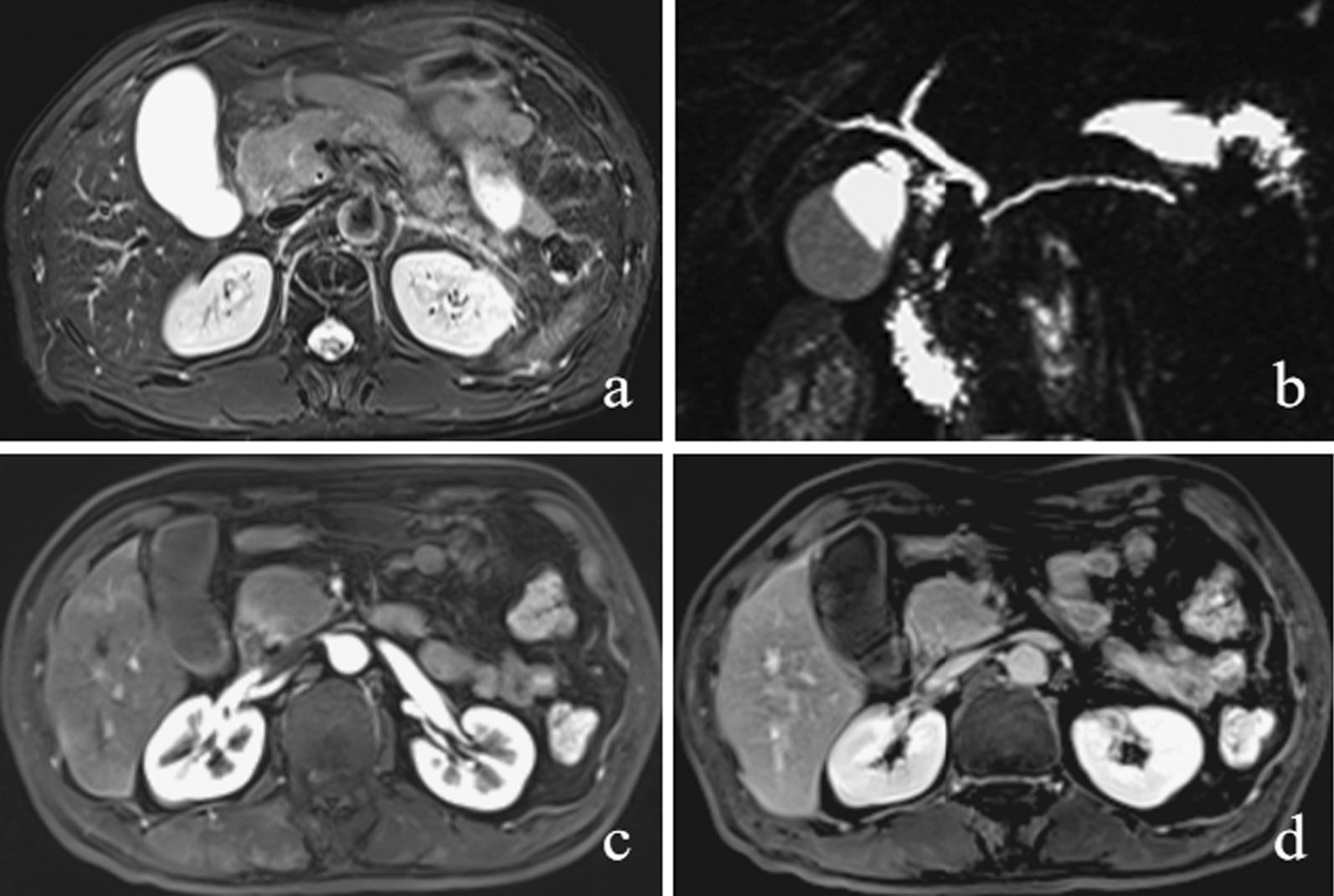
A 57-year-old man had PDAC. **a** T2WI, the round pancreatic head mass shows hyperintensity with homogeneity. **b** MRCP image shows the cut-off of the CBD and MPD. On the PP image (**c**) shows the mass with hypointensity. **d** On the DP image, the mass shows homogeneous enhancement

According to dynamic imaging, several AIP and PDAC lesions showed hypointense and heterogeneous enhancement during the AP, albeit no significant difference was detected between AIP and PDAC groups (*P* > 0.05). However, PDAC more frequently presented a heterogeneous enhancement (Figs. 4, 5), while AIP displayed a homogeneous enhancement on PP/VP/DP (*P* ≤ 0.002). Mass-forming AIP frequently presented hyperintensity as compared with PDAC on PP/VP/DP (*P* ≤ 0.017).

### Mass enhancement contrast

Table 3 summarizes the mass enhancement contrast results. Quantitative analysis showed that the mass enhancement contrast differed significantly between AIP and PDAC groups at all phases of DCE-MRI (*P* ≤ 0.003). In AIP, the lesion contrast which was the lowest at the unenhanced phase, increased gradually at the later phases. The contrast ultimately rose to > 1.0 at PP (9/17, 0.53%), indicating AIP had high SI in contrast with NAP. In PDAC, the contrast was the lowest at the AP phase. It decreased from the unenhanced phase to the AP but progressively increased thereafter. However, the contrast remained > 1.0 in most lesions (14/17, 82.4%) at the DP compared with PDAC. The VP was the maximum difference phase of the lesion contrast between AIP and PDAC. Sensitivity and specificity were 88.2% and 70.0%, respectively, when the cutoff value for the Contrast_VP_ value was 0.945 for differentiating AIP from PDAC. The AUROC curve was 0.859.

**Table 3:** Quantitative analysis of the mass enhancement contrast

	AIP (*N* = 17)	PDAC (*N* = 30)	*P* value	ACROC*	*P* value^#^
Contrast_UP_, mean (range, SD)	0.79 (0.55–1.06, 0.16)	0.66 (0.44–0.98, 0.11)	0.003	0.735 (0.579–0.892)	0.172
Contrast_AP_, mean (range, SD)	0.85 (0.45–1.18, 0.21)	0.55 (0.22–1.16, 0.19)	< 0.001	0.851 (0.734–0.968)	0.885
Contrast_PP_, mean (range, SD)	1.10 (0.73–1.63, 0.26)	0.70 (0.21–1.38, 0.28)	< 0.001	0.851 (0.745–0.957)	0.733
Contrast_VP_, mean (range, SD)	1.28 (0.83–1.92, 0.34)	0.79 (0.24–1.48, 0.30)	< 0.001	0.859 (0.755–0.963)	
Contrast_DP_, mean (range, SD)	1.30 (0.83–1.79, 0.28)	1.00 (0.30–1.53, 0.33)	0.003	0.749 (0.607–0.891)	0.009

*UP* unenhanced phase, *AP* arterial phase, *PP* pancreatic phase, *VP* portal venous phase, *DP* delayed phase

*AUROC for differentiating between AIP and PDAC (95% CI)

^#^Compared to AUROC of Contrast_VP_

### MRCP findings

Regarding the MRCP features of AIP and PDAC, no significant differences were detected in the CBD abnormalities and penetrating duct sign. MPD stricture was more commonly detected in AIP than PDAC (*P* = 0.001). Furthermore, significant differences were also observed in skipped strictures of the MPD (3/12, 25%) and the side branch dilation (1/12, 8.3%) (*P* = 0.019, *P* = 0.004, respectively). Only one AIP patient presented a dilated side branch of the pancreatic duct, whereas skipped strictures of the MPD were seen exclusively in AIP patients. However, the maximum diameter of the upstream MPD was not available for masses located in pancreatic tail, while that of the upstream MPD of 26 masses of PDAC [mean ± standard deviation (SD), 5.1 ± 2.4; range: 1.0–9.6 mm] was significantly larger than that of 9 masses of AIP (1.9 ± 0.7; range: 1.2–3.5 mm). When the cutoff value for the maximum diameter of the upstream MPD was set at 2.4 mm for distinguishing AIP from PDAC, a sensitivity of 88.9%, specificity of 84.6%, and an AUROC curve of 0.859 were obtained. The MRCP features are listed in Table 4.

**Table 4:** MRCP findings between AIP and PDAC

MRCP findings	AIP (*N* = 12)	*k*	PDAC (*N* = 30)	*k*	*P* value
CBD		0.867		0.795	0.570
Normal	6		13		
Stricture	4		7		
Complete obstruction	2		10		
CBD skipped strictures	1	1	0	1	0.286
MPD		0.871		0.927	0.001
Normal	0		4		
Stricture	6		1		
Complete obstruction	3		21		
Not visible	3		4		
MPD skipped strictures	3	0.750	0	1	0.019
Side branch dilation	1	1	17	0.733	0.004
Penetrating duct sign	2	1	0	1	0.077
Maximum upstream MPD diameter (mm)*	1.9 ± 0.7 (1.2–3.5)	–	5.1 ± 2.4 (1.0–9.6)	–	< 0.001

Numbers are the number of patients

*Masses located on the edge of the pancreatic tail were excluded in 4 AIP patients. Data are means ± standard deviation with the ranges in parentheses

### Quantitative analysis of DWI

The results of the DWI findings are shown in Table 5. The mean ADC value (× 10^−3^ mm^2^/s) of the masses was lower in mass-forming AIP than PDAC (1.020 ± 0.119, 1.158 ± 0.208, respectively; *P* = 0.006), while no statistical difference was revealed in NAP between AIP and PDAC at DWI (1.246 ± 0.185, 1.233 ± 0.166, respectively; *P* = 0.818). Also, there is no significant difference in the normalized ADC between AIP and PDAC (*P* = 0.048). When the cutoff value for the ADC was set at 1.099 × 10^−3^ mm^2^/s in the ROC curve analysis for differentiation between the two groups, the sensitivity and specificity were 88.2% and 60.0%. An AUROC curve of 0.733 was obtained. In addition, the mean diameter AIP was 34.5 ± 24.3 (range: 10.0–97.0) mm, which did not differ significantly from that of PDAC (33.5 ± 13.4 mm, 15.7–70.1 mm) (*P* = 0.877).

**Table 5:** Results of ADC value and normalized ADC

	AIP	PDAC	*P* value
Diameter (mm)	34.5 ± 24.3	33.5 ± 13.4	0.877
Mass ADC value (× 10^−3^ mm^2^/s)	1.020 ± 0.119	1.158 ± 0.208	0.006
NAP ADC value (× 10^−3^ mm^2^/s)	1.246 ± 0.185	1.233 ± 0.166	0.818
Normalized ADC*	0.838 ± 0.130	0.953 ± 0.205	0.048

Data are means ± standard deviation in parentheses; NAP, non-mass adjacent pancreatic parenchyma; Normalized ADC, pancreatic mass to non-mass adjacent pancreatic parenchymal ADC ratio. Normalized ADC* was available in 16 lesions of AIP and in 30 lesions of PDAC

Table 6 shows the sensitivity and specificity for imaging findings. The venous phase homogeneous enhancement achieved a relatively high sensitivity of 70.6% and specificity of 93.3%. The absence of internal cystic or necrotic portion had the highest sensitivity of 100.0%, while the specificity was 33.3%. The multiplicity, capsule-like rim enhancement and MPD skipped strictures had maximal specificity of 100.0%, while the sensitivity was low.

**Table 6:** Sensitivity and specificity of the significant MR findings in the diagnosis of mass-forming AIP

	Sensitivity (%)	Specificity (%)
Multiplicity	33.3 (4/12)	100 (28/28)
Irregular or geographical shape	52.9 (9/17)	90 (27/30)
Capsule-like rim enhancement	35.3 (6/17)	100 (30/30)
Absence of internal cystic or necrotic portion	100 (17/17)	33.3 (10/30)
Absence of side branch dilation	91.7 (11/12)	56.7 (17/30)
Pancreatic phase homogeneous enhancement	47.1 (8/17)	93.3 (28/30)
Venous phase homogeneous enhancement	70.6 (12/17)	93.3 (28/30)
Delayed phase homogeneous enhancement	76.5 (13/17)	93.3 (28/30)
MPD stricture	50.0 (6/12)	96.7 (29/30)
MPD skipped strictures	25.0 (3/12)	100 (30/30)
Maximum upstream MPD diameter < 2.4 mm	88.9 (8/9)	84.6 (22/26)
ADC value < 1.099 × 10^−3^ mm^2^/s)	88.2 (15/17)	60.0 (18/30)
Contrast_UP_ > 0.737	64.7 (11/17)	83.3 (25/30)
Contrast_AP_ > 0.690	82.4 (14/17)	83.3 (25/30)
Contrast_PP_ > 0.862	82.4 (14/17)	76.7 (23/30)
Contrast_VP_ > 0.945	88.2 (15/17)	70.0 (21/30)
Contrast_DP_ > 0.942	88.2 (15/17)	50.0 (15/30)

The data in parentheses represent the number of patients or masses

## Discussion

The imaging characteristics of mass-forming AIP using multimodel magnetic resonance imaging are critical for differentiation between mass-forming AIP and PDAC as misdiagnosis could lead to unnecessary invasive and surgical procedures [15–18].

The current study showed several statistically significant MR findings and quantitative indexes discriminating between the two conditions, and including multiplicity, irregular or geographical morphology, capsule-like rim enhancement, absence of internal cystic or a necrotic portion, homogeneous enhancement during the PP/VP/DP, skipped stricture or stricture of MPD, absence of side branch dilation, maximum upstream MPD diameter < 2.4 mm, ADC value < 1.099 × 10^−3^ mm^2^/s, Contrast_UP_ > 0.739, Contrast_AP_ > 0.710, Contrast_PP_ > 0.879, and Contrast_VP_ or Contrast_DP_ > 0.949; mass-forming AIP was more likely to exhibit these parameters together. Furthermore, AIP had the highest specificity in the imaging characteristics of multiplicity, capsule-like rim, and skipped stricture of MPD and hence, the possibility of PDAC could be excluded. Alternatively, internal cystic or a necrotic portion, side branch dilation, and heterogeneous enhancement during the PP/VP/DP suggested PDAC. Herein, 7 cases of mass-forming AIP were diagnosed by biopsy or even surgery. Based on the analysis of multimodel MRI findings, we had reason to conclude that our results were crucial for avoiding invasive or surgical procedures in mass-forming AIP that could be easily differentiated from PDAC.

One of representative imaging characteristics of AIP is the capsule-like rim that is used to distinguish AIP from PDAC. However, in recent studies, the sensitivity of the capsule-like rim sign with focal or massing AIP was low at 19.2–47.5% [19–22]. In our study, this feature was observed in 6/17 (35.3%) masses with AIP; however, the major drawback was poor sensitivity. Similarly, multiplicity had high specificity (100.0%) but poor sensitivity (33.3%). Furthermore, the existence of multiple pancreatic masses can reflect the fact that the focal inflammatory masses can affect multiple areas of the pancreas. Moreover, multimodel MRI findings include not only morphological and signal features of conventional MRI but also DCE-MRI and DWI, which has an outstanding ability to detect multiple masses, especially a subtle lesion. Typically, the pancreatic mass observed in AIP showed an irregular or geographical morphology, unlike the oval or round shape observed in PDAC.

Our study detected that quantitative analysis of the mass enhancement contrast using DCE-MRI could be valuable for differentiating between the two conditions. Furthermore, the quantitative analysis results suggested that Contrast_VP_ can be used as a vital index in the differentiation between AIP and PDAC in view of its excellent diagnostic performance when compared with other phase indexes. Kwon et al. [22] reported that Contrast_AP_ had excellent diagnostic performance for differentiating between the two diseases which was in disaggrement with our study. We assume that this discrepancy may arise from difference in MRI parameters or in criteria for inclusion of the study population. We analyzed all lesions, including small ones. Interestingly, we observed the homogenous enhancement on PP which was significantly more frequent in mass-forming AIP than in PDAC. These results suggested that AIP with multiple masses showed early homogeneous enhancement, which could be attributed to the small size of some lesions and focal inflammation. Previous studies had reported the key imaging feature of delayed homogeneous enhancement in mass-forming AIP [9, 20–25], which was consistent with the current findings. Pathologically, both AIP and PDAC presented abundant fibrous stroma, which usually resulted in delayed enhancement. However, heterogeneous enhancement of the lesions of PDAC has frequently been observed on DCE-MRI because of necrosis, cyst degeneration, or hemorrhage [26–28].

In this study, the mean ADC value of masses is lower in AIP than in PDAC, while no statistical difference was observed in the SI on DWI, NAP ADC value, and the normalized ADC between the two conditions. When the ADC cutoff value in distinguishing mass-forming AIP from PDAC was 1.099 × 10^−3^ mm^2^/s, a sensitivity of 88.2% and specificity of 60.0% was obtained. Several studies reported different ADC optimal cutoff values, ranging from 0.88–1.26 × 10^−3^ mm^2^/s, so as to differentiate mass-forming AIP from PDAC. Our cutoff value of ADC is in the range reported in previous studies, although our specificity was the lowest [1, 9, 20, 29]. However, the reason why mass-forming AIP had a lower mean ADC value than PDAC is yet to be elucidated. We presume that the lymphoplasmacytic infiltration and fibrous tissue in AIP is more abundant than that of PDAC, which includes diffuse infiltration of cancer cells and abundant fibrosis component [29]. Increased cellularity, chronic fibroinflammatory processes, and pancreatic tissue edematous changes may play critical roles in achieving the lower ADC values in mass-forming AIP than in PDAC [29]. In addition, a common internal cystic or necrotic portion in PDAC was responsible for obtaining high ADC values [1, 29]. These factors may contribute to the difference in ADC value observed in this study between the two groups. Together, the current and previous results [1, 9, 20] have demonstrated that DWI is vital for the detection of mass-forming AIP especially multifocal or small lesions as well as the differentiation of AIP from PDAC.

In this study, only AIP showed skipped strictures of MPD, which might be an indicator of mass-forming AIP that presented skipped MPD narrowing, less complete obstruction MPD, less dilated upstream MPD, and less side branch dilation on MRCP. These results were consistent with those reported previously [8, 19, 20]. However, there were no statistically significant difference between mass-forming AIP and PDAC regarding CBD abnormalities or penetrating duct sign. As reported in the literature, a prevalence of skipped strictures of MPD was observed to be developed along with the whole extension of the MPD in the patients with multiple stenoses even if the lesions involved were segmental [19]. This finding of skipped strictures of MPD may reflect the extension of inflammatory infiltration which can lead to focal enlargement of the pancreas. The inflammatory compression of AIP leads to a mild dilation of the MPD upstream dilatation (mean = 2.4 mm in our study) unlike PDAC, in which CBD or pancreatic duct abnormalities are induced by cancer cell infiltrating the ductal system; consequently, MPD cutoff or marked dilation of the upstream MPD or side branch dilation were also observed. Previous study revealed that administration of secretin in AIP could reduce number and length of MPD stenoses [20, 30, 31]. Secretin administration enhances the performance of MRCP, especially the evidence of penetrating duct sign. Based on these results, adding MRCP to conventional MRI and DCE-MRI would be significant for diagnosing AIP as it provides additional information of CBD and MPD.

Nevertheless, this study has some limitations. First, the possibility of selection bias due to the retrospective study design should be considered. Second, the number of patients with mass-forming AIP was limited, and only 12 patients were available for MRI examination. Third, the lesion size was small in some cases of mass-forming AIP, which might have biased the determination of signal intensity qualitatively and quantitatively.

## Conclusions

We identified characteristic MR findings that can differentiate mass-forming from PDAC. Multimodel MRI, including unenhanced MRI, DCE-MRI with DWI and MRCP can provide qualitative and quantitative information about mass-forming AIP characterization. Multimodel MRI can be helpful for the appropriate diagnosis of mass-forming AIP.

## Data Availability

The datasets analyzed in this study are available from the corresponding author on request.

## Abbreviations

MRI: Magnetic resonance imaging
DCE-MRI: Dynamic contrast-enhanced MRI
MRCP: MR-cholangiopancreatography
DWI: Diffusion-weighted imaging
AIP: Autoimmune pancreatitis
PDAC: Pancreatic ductal adenocarcinoma
